# Supplementary material for: Widespread Autonomic Physiological Coupling Across the Brain-Body Axis
Source: bioRxiv. 2024 Jul 29:2023.01.19.524818. Preprint. [Version 3] doi: 10.1101/2023.01.19.524818 (PMC11312447; doi:10.1101/2023.01.19.524818)
Supplement: Supplement 1 [file NIHPP2023.01.19.524818v3-supplement-1.pdf]

bioRxiv preprint doi: https://doi.org/10.1101/2023.01.19.524818; this version posted July 29, 2024. The copyright holder for this preprint (which was not certified by peer review) is the author/funder, who has granted bioRxiv a license to display the preprint in perpetuity. It is made available under aCC-BY 4.0 International license.

Supplementary Materials

Supplementary Movies

Supplementary Movie 1. **Temporal Reconstruction of CPC1 in ME-REST Dataset.**

Supplementary Movie 2. **Temporal Reconstruction of CPC1 in ME-REST-SUPP Dataset.**

bioRxiv preprint doi: https://doi.org/10.1101/2023.01.19.524818; this version posted July 29, 2024. The copyright holder for this preprint (which was not certified by peer review) is the author/funder, who has granted bioRxiv a license to display the preprint in perpetuity. It is made available under aCC-BY 4.0 International license.

Supplementary Movie 3. **Cross-Correlation of fMRI Signals and PPG Amplitude in ME-REST Dataset.**

Supplementary Movie 4. **Cross-Correlation of fMRI Signals and PPG Amplitude in ME-REST-SUPP Dataset.**

Supplementary Tables

Dataset	Sample	Physio Signals Used	Source	Sample Size	Number of Sessions	Age Range	Sex (F)
ME-REST	Full	EEG, Respiration, PPG	Goodale et al. (2021). *ELife*. https://doi.org/10.7554/eLife.62376	11	15	21-35	6
ME-TASK	Full	EEG, Respiration, PPG	Unpublished dataset; https://www.cchanglab.net/	6	9	22-57	4
ME-TASK-CUE	Full	EEG, Respiration, PPG	Goodale et al. (2021). *ELife*. https://doi.org/10.7554/eLife.62376	12	12	21-33	6
ME-REST-SUPP	Subset	Respiration, PPG	https://openneuro.org/datasets/ds003592/versions/1.0.11 **OpenNeuro Accession Number:** ds003592 **Version:** 1.0.11	87	165	18 - 34	58
HCP-REST	Subset	Respiration, PPG	https://www.humanconnectome.org/	30	30	22-37	17
NKI-TASK	Subset	Respiration, PPG, Skin Conductance	http://fcon_1000.projects.nitrc.org/indi/enhanced/	50	50	15-45	30

bioRxiv preprint doi: https://doi.org/10.1101/2023.01.19.524818; this version posted July 29, 2024. The copyright holder for this preprint (which was not certified by peer review) is the author/funder, who has granted bioRxiv a license to display the preprint in perpetuity. It is made available under aCC-BY 4.0 International license.

NKI-REST	Subset	Respiration, PPG, Skin Conductance	http://fcon_1000.projects.nitrc.org/indi/enhanced/	50	50	18-45	33
NATVIEW-REST	Full	EEG, Pupillometry	(Telesford et al., 2023)	21	33	22-51	10
YALE-REST	Full	Pupillometry	https://openneuro.org/datasets/ds003673/versions/2.0.1<br><br>**OpenNeuro Accession Number:** ds003673 **Version:** 2.0.1	27	54	21-37	16
Clamped CO2	Full	N/A	(Golestani & Chen, 2020)	13	13	18-32	9

Supplementary Table 1. **Dataset Details and Demographics.** Details (manuscript label, signals recorded, reference) and demographics (sample size, age range, sex) for datasets used in this study. Note, demographics are based on the dataset sample after the quality control stage.

Supplementary Figures

bioRxiv preprint doi: https://doi.org/10.1101/2023.01.19.524818; this version posted July 29, 2024. The copyright holder for this preprint (which was not certified by peer review) is the author/funder, who has granted bioRxiv a license to display the preprint in perpetuity. It is made available under aCC-BY 4.0 International license.

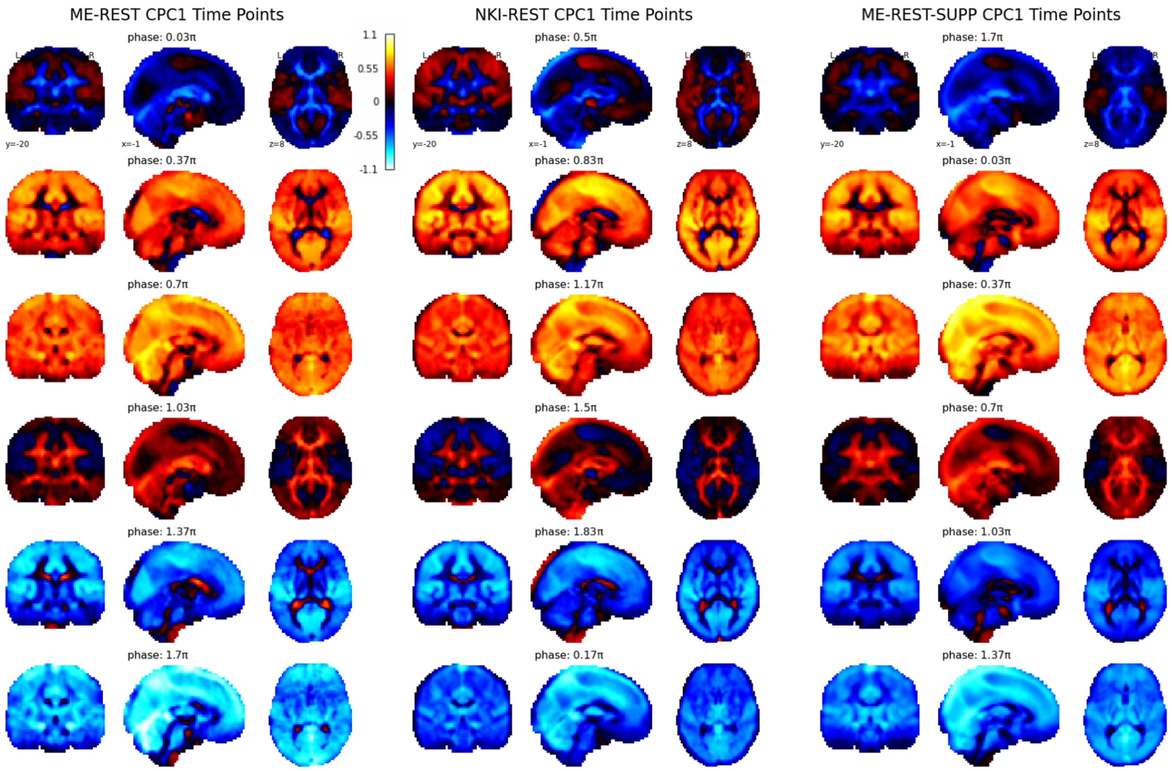

Supplementary Figure 1. **Spatiotemporal Dynamics of Global fMRI Signals.** Temporal reconstruction of the first complex principal component (CPC1) of three resting-state fMRI datasets (ME-REST, NKI-REST, ME-REST-SUPP). Complex PCA (CPCA) on other resting-state datasets (HCP-REST, NATVIEW-REST, YALE-REST) yielded similar results, and are not included here for space. As shown in previous work, the first principal component from PCA and complex principal component from CPCA extract the global fMRI signal, with CPCA yielding a spatiotemporal representation of the global fMRI signal (Bolt et al., 2022). The time course of a complex principal component is represented in complex numbers, and its phase can be extracted (measured in radians). A temporal reconstruction of the complex principal component can be constructed via averaging of the original fMRI signals (in voxel space) at similar phase values. We selected six equally spaced phase values to display the spatiotemporal dynamics of the global fMRI signal. Time moves in the positive direction, such that increasing phase values move forward in time. A consistent spatiotemporal pattern is observed across datasets: a global increase in fMRI signals in the gray and white matter followed by a propagation of fMRI signals to large draining veins and ventricles, and then a global decrease in fMRI signals.

bioRxiv preprint doi: https://doi.org/10.1101/2023.01.19.524818; this version posted July 29, 2024. The copyright holder for this preprint (which was not certified by peer review) is the author/funder, who has granted bioRxiv a license to display the preprint in perpetuity. It is made available under aCC-BY 4.0 International license.

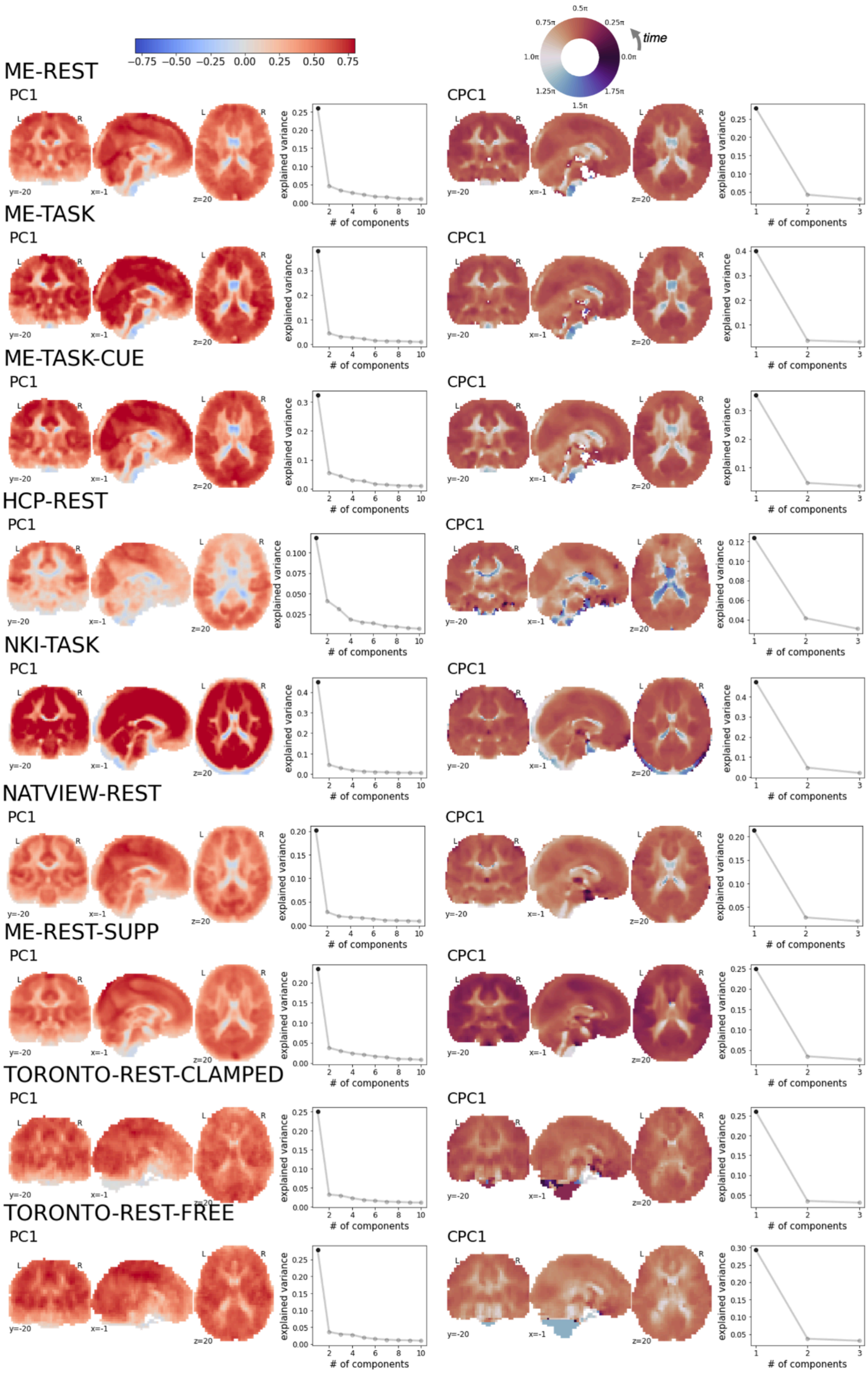

bioRxiv preprint doi: https://doi.org/10.1101/2023.01.19.524818; this version posted July 29, 2024. The copyright holder for this preprint (which was not certified by peer review) is the author/funder, who has granted bioRxiv a license to display the preprint in perpetuity. It is made available under aCC-BY 4.0 International license.

Supplementary Figure 2. **First Principal Component and Complex Principal Component Across Datasets.** Spatial weights of the first principal component (PC1; left) and phase delay maps of the first complex principal component (CPC1; right) across all datasets used in this study. Explained variance plots (Scree plots) are displayed to the right of each brain map displaying the explained variance by the first and subsequent principal components. The phase delay map of the first complex principal component encodes the time-delay (in radians) between voxels within the component. Because phase delay is measured in radians (0 to 2pi), they are displayed with a circular color map.

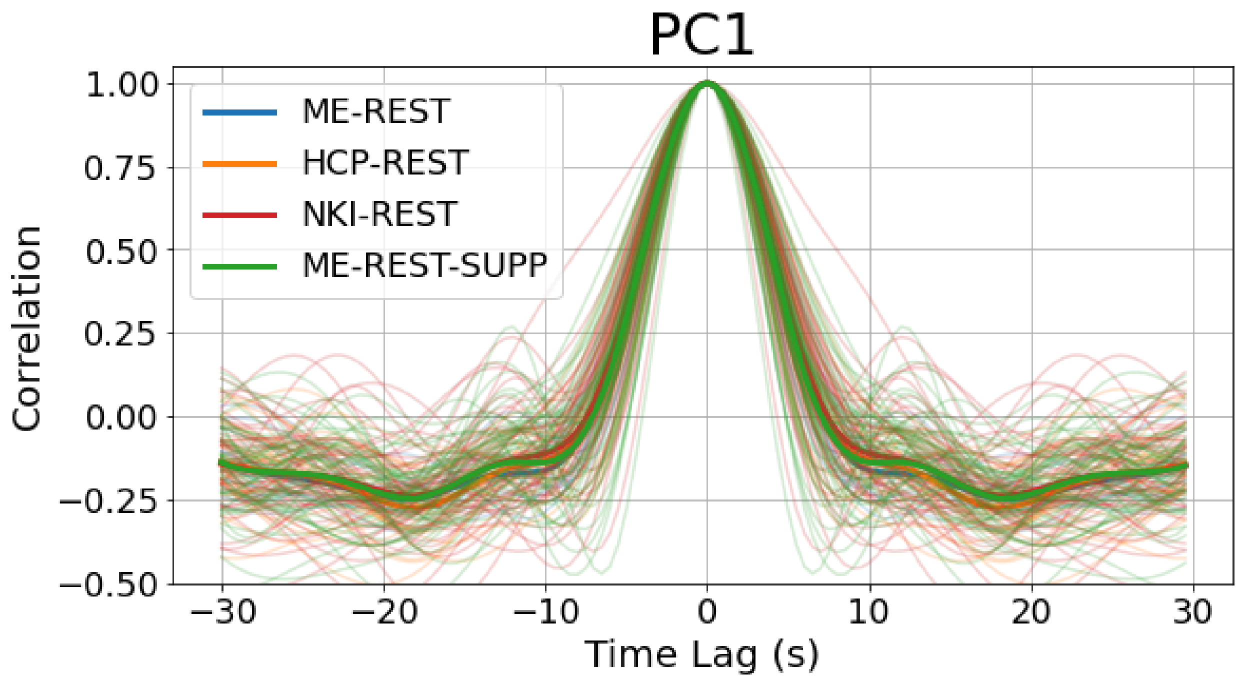

Supplementary Figure 3. **Auto-Correlation Plot of PC1 Time Courses.** The cross-correlation of the PC1 time course with itself from the ME-REST, HCP-REST, NKI-REST and ME-REST-SUPP datasets. Subject-level auto-correlations are displayed in lighter colors, and the group-average auto-correlation is displayed with a thicker, darker line.

bioRxiv preprint doi: https://doi.org/10.1101/2023.01.19.524818; this version posted July 29, 2024. The copyright holder for this preprint (which was not certified by peer review) is the author/funder, who has granted bioRxiv a license to display the preprint in perpetuity. It is made available under aCC-BY 4.0 International license.

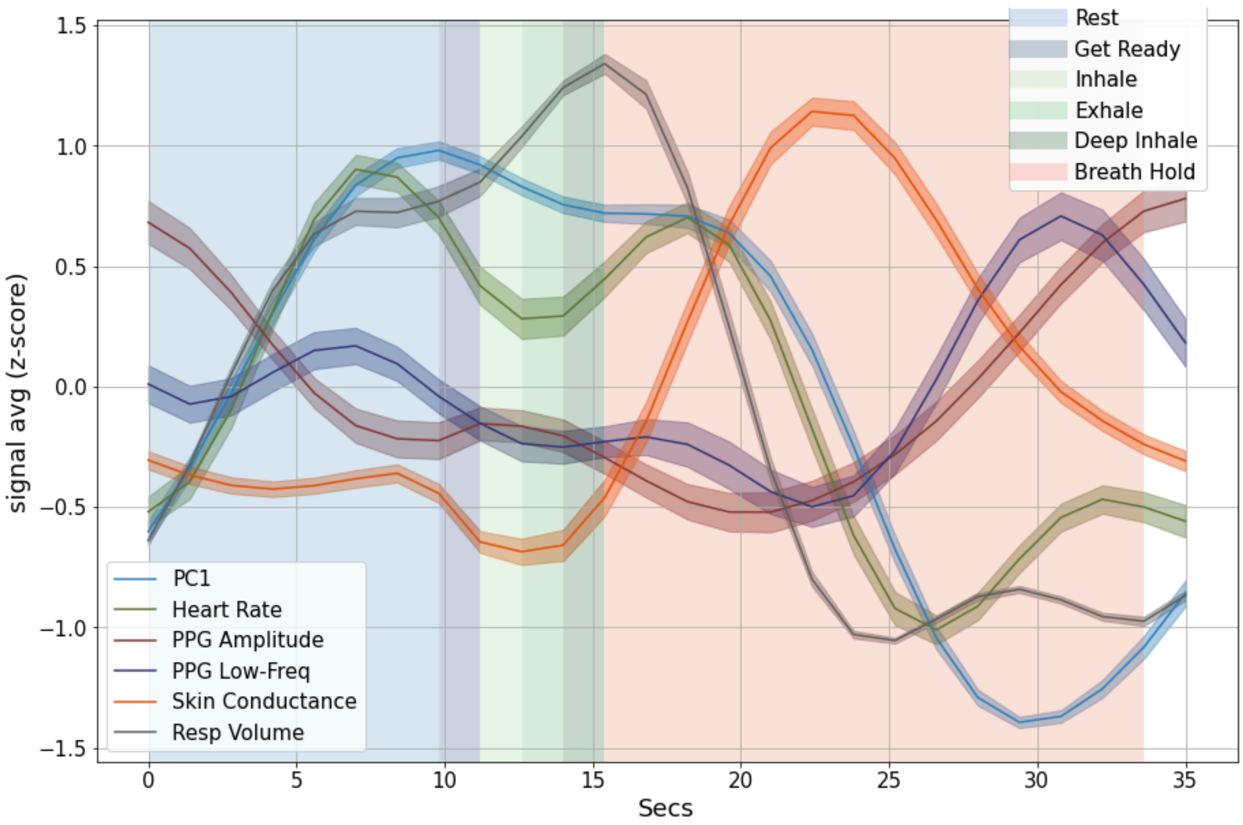

Supplementary Figure 4. **NKI-Breath Hold Task.** Physiological trial averages for a full block of the paced inhalation/breath hold task (NKI-TASK). The paced breathing/breath-hold task was a block design consisting of a fixed sequence of rest (10s), a cue (2s) followed by two deep inhalations (6s), and a breath-hold (18s), immediately followed by another sequence. Time points in separate blocks are shaded to distinguish activity in each block: rest (10s duration; blue), ‘get ready’ cue (2s; dark blue), inhalation (2s; light green), exhalation (2s; medium green), deep inhalation (2s; dark green) and breath hold (18s; red).The physiological dynamics of the paced breathing/breath hold task (NKI-TASK) are more complex than those observed in response to isolated deep inhalations (**Figure 2A**). For example, large increases in respiratory volume are observed during the paced inhalation exercise before the breath hold and the ‘rest’ blocks that precede the paced inhalation exercise, due to their placement immediately after the breath hold. The timing of the large amplitude peak of global fMRI signals in the rest period (~10s) and following the deep inhalation block (19s) are consistent with the timing of the respiration response peak observed to isolated deep inhalations (**Figure 2A**). Consistent with the response to isolated deep inhalations, an increase in heart rate is observed around the time of the global fMRI peak, shortly followed by peripheral vasoconstriction. In addition, a large amplitude response in skin conductance is observed around the same time as peripheral vasoconstriction. The trough of the global fMRI response, along with peripheral vasodilation, occurs in the latter half of the breath hold block, consistent with the peak-to-trough timing observed in deep inhalations.

bioRxiv preprint doi: https://doi.org/10.1101/2023.01.19.524818; this version posted July 29, 2024. The copyright holder for this preprint (which was not certified by peer review) is the author/funder, who has granted bioRxiv a license to display the preprint in perpetuity. It is made available under aCC-BY 4.0 International license.

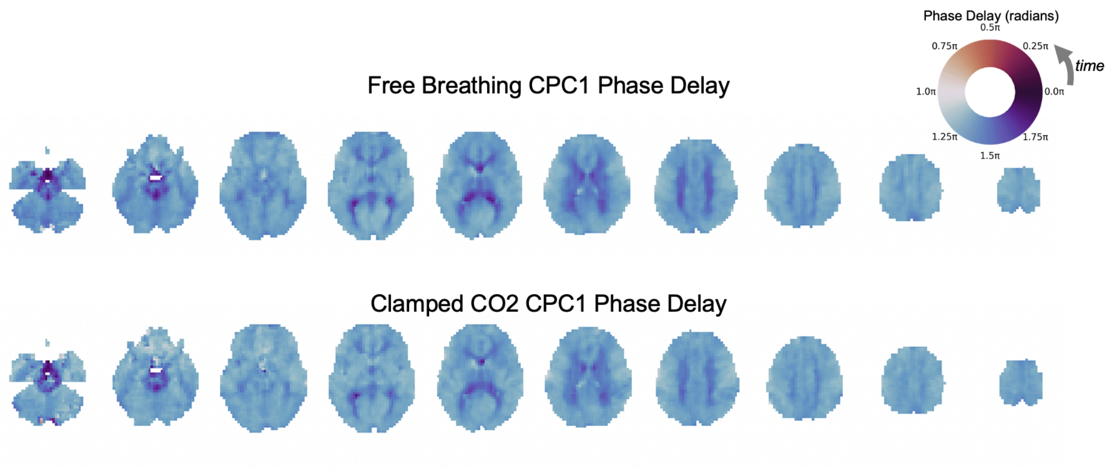

Supplementary Figure 5. **First Complex Principal Component Phase Delay in Free Breathing and Clamped CO2 Conditions.** Phase delay maps of the first complex principal component (CPC1) computed from fMRI time courses in free breathing and clamped CO2 conditions. The phase delay map of the first complex principal component encodes the time-delay (in radians) between voxels within the component. Because phase delay is measured in radians (0 to 2pi), they are displayed with a circular color map. As can be observed from the brain maps, the distribution of phase delay values across the brain is highly similar across free breathing conditions and clamped conditions.

bioRxiv preprint doi: https://doi.org/10.1101/2023.01.19.524818; this version posted July 29, 2024. The copyright holder for this preprint (which was not certified by peer review) is the author/funder, who has granted bioRxiv a license to display the preprint in perpetuity. It is made available under aCC-BY 4.0 International license.

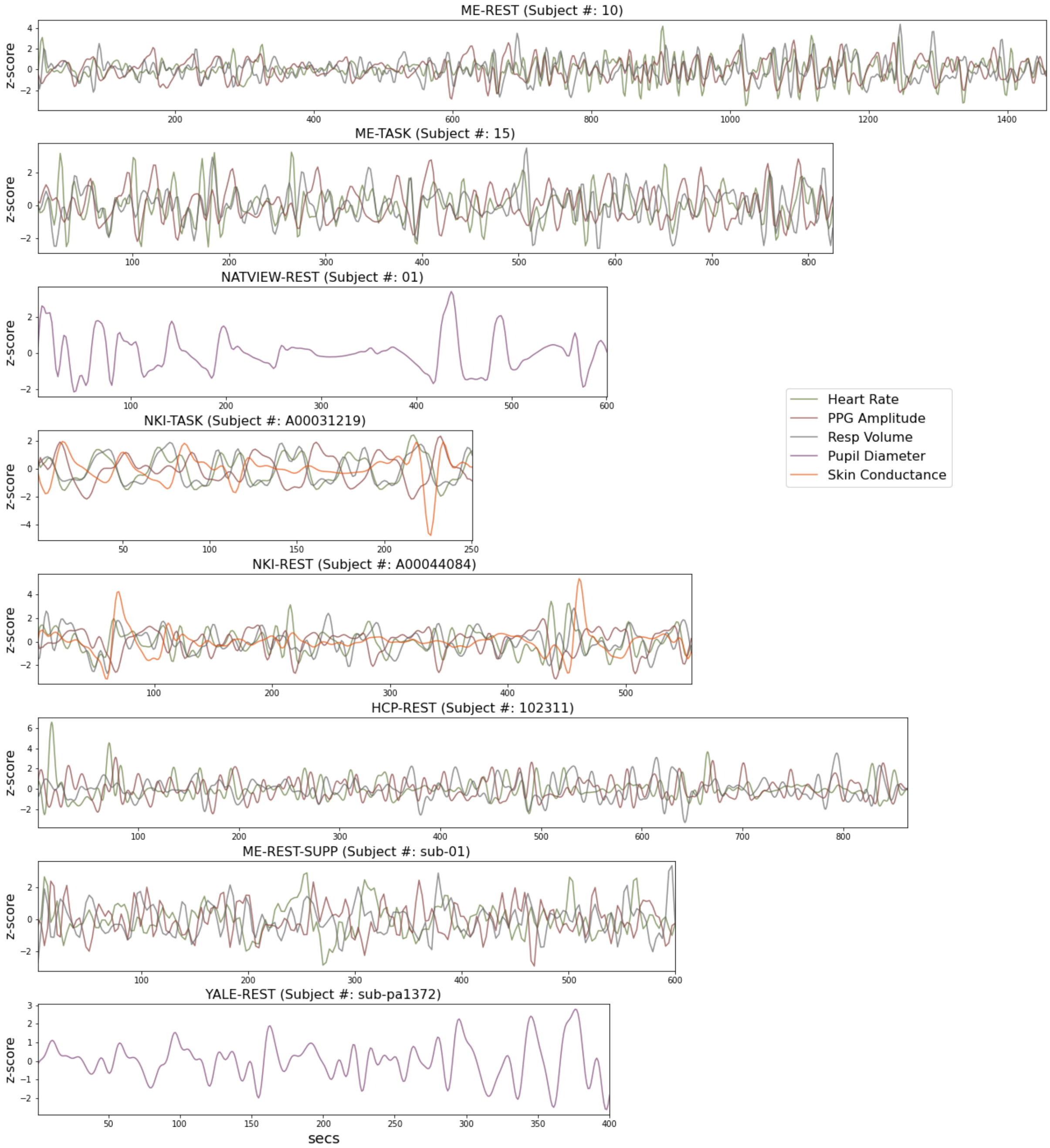

Supplementary Figure 6. **Physiological Time Series from Example Subjects.**

bioRxiv preprint doi: https://doi.org/10.1101/2023.01.19.524818; this version posted July 29, 2024. The copyright holder for this preprint (which was not certified by peer review) is the author/funder, who has granted bioRxiv a license to display the preprint in perpetuity. It is made available under aCC-BY 4.0 International license.

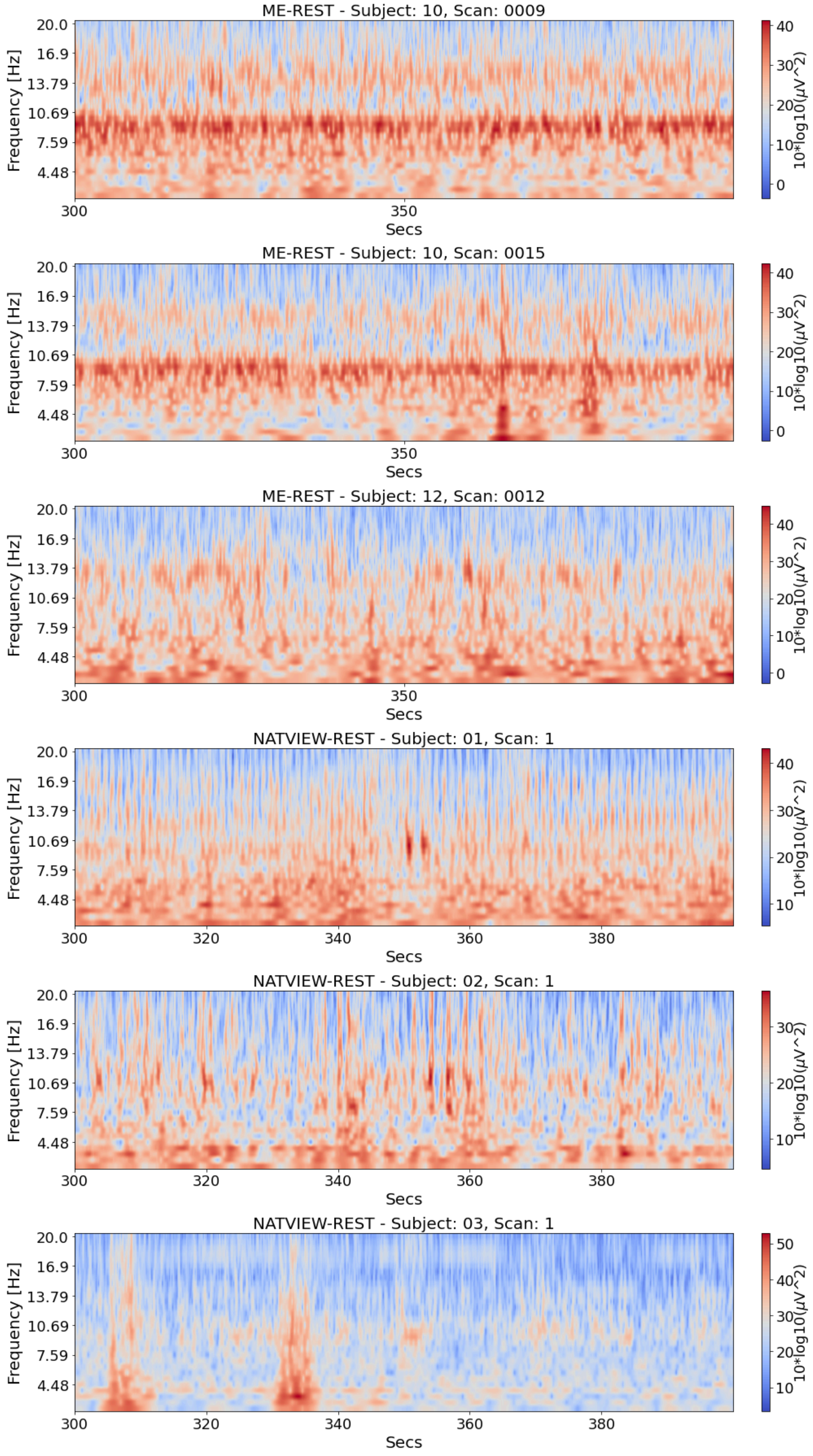

bioRxiv preprint doi: https://doi.org/10.1101/2023.01.19.524818; this version posted July 29, 2024. The copyright holder for this preprint (which was not certified by peer review) is the author/funder, who has granted bioRxiv a license to display the preprint in perpetuity. It is made available under aCC-BY 4.0 International license.

Supplementary Figure 7. **Channel-Averaged EEG Spectrograms Samples from Example Subjects.** EEG time-frequency power plots of sample time points (300 - 400 seconds post scan-onset) averaged across channels used in the study (posterior and occipital channels). Time-frequency EEG power was extracted via Morlet wavelet filters using the same parameters for analyses presented in **Figure 1** and **Figure 2** (number of cycles = 15; frequencies: 2 - 20Hz).

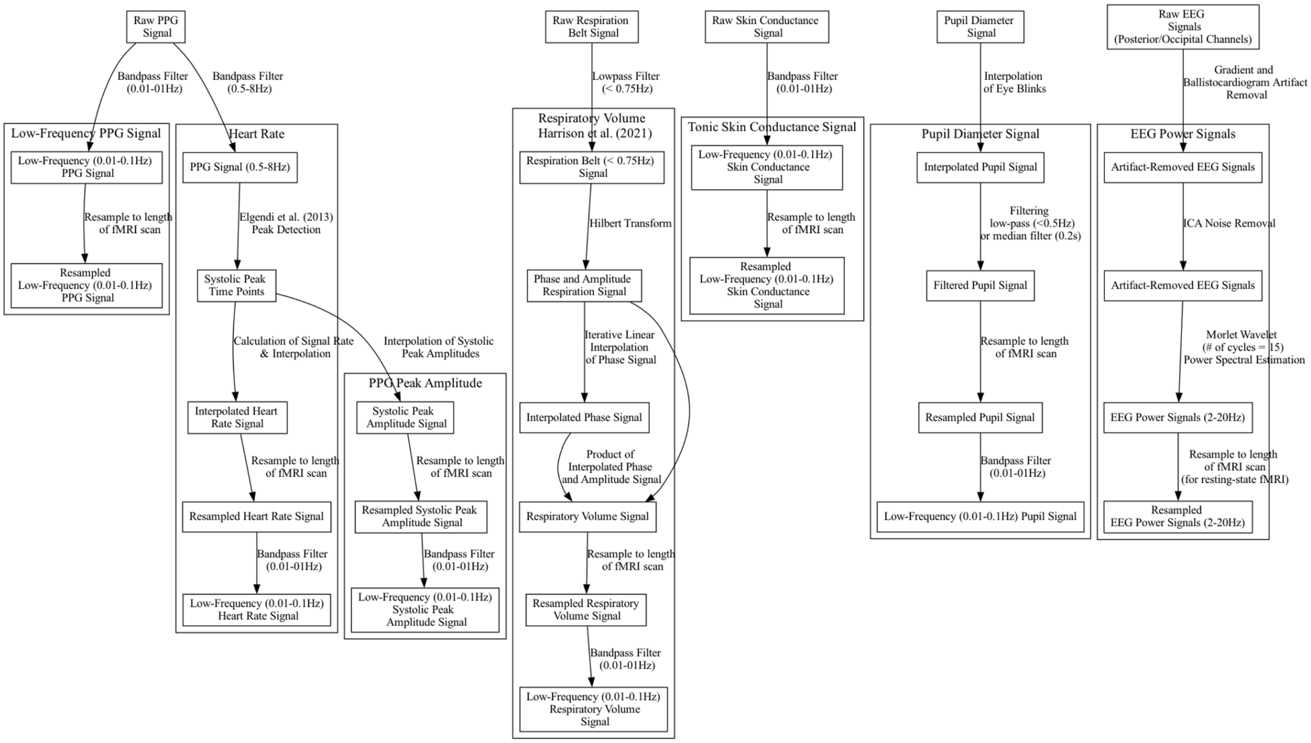

Supplementary Figure 8. **Electrophysiological Signal Preprocessing Pipeline.**

bioRxiv preprint doi: https://doi.org/10.1101/2023.01.19.524818; this version posted July 29, 2024. The copyright holder for this preprint (which was not certified by peer review) is the author/funder, who has granted bioRxiv a license to display the preprint in perpetuity. It is made available under aCC-BY 4.0 International license.

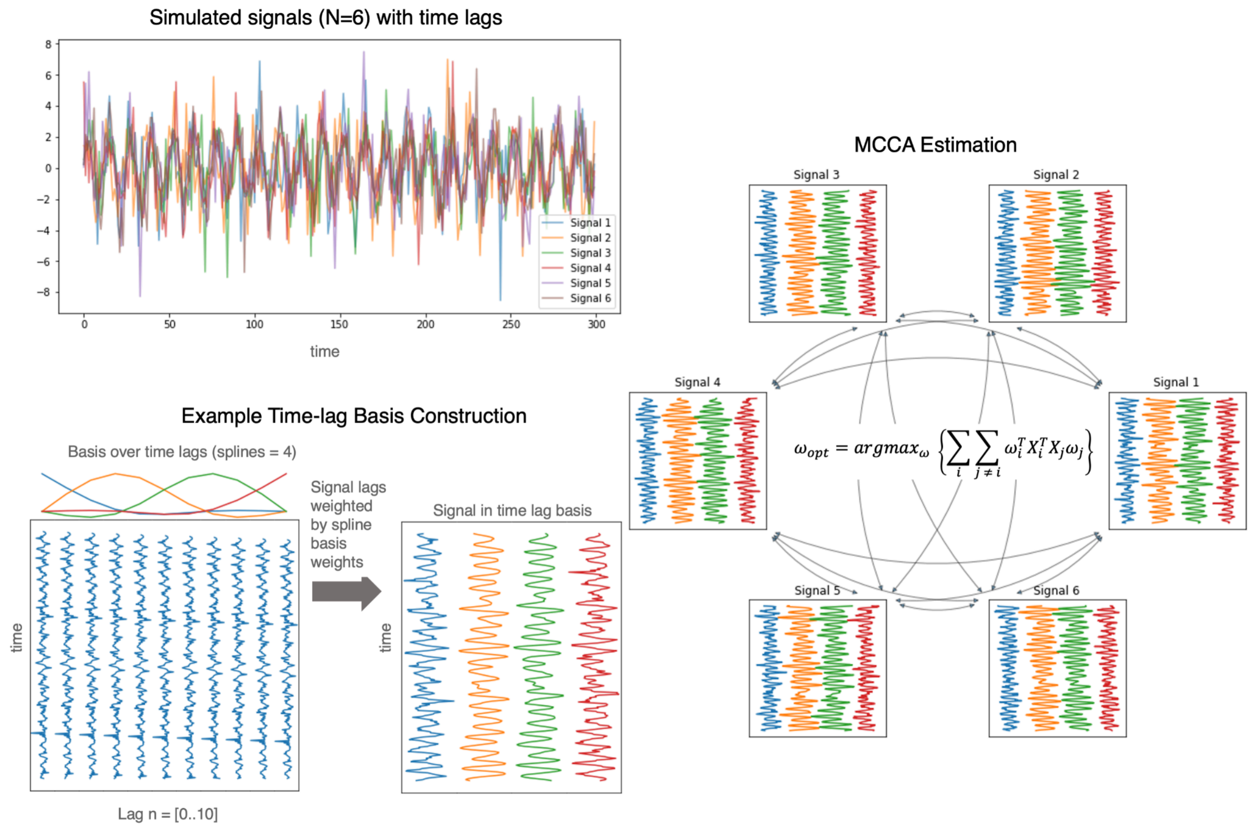

Supplementary Figure 9. **Illustration of Multiset Canonical Correlation Analysis.**

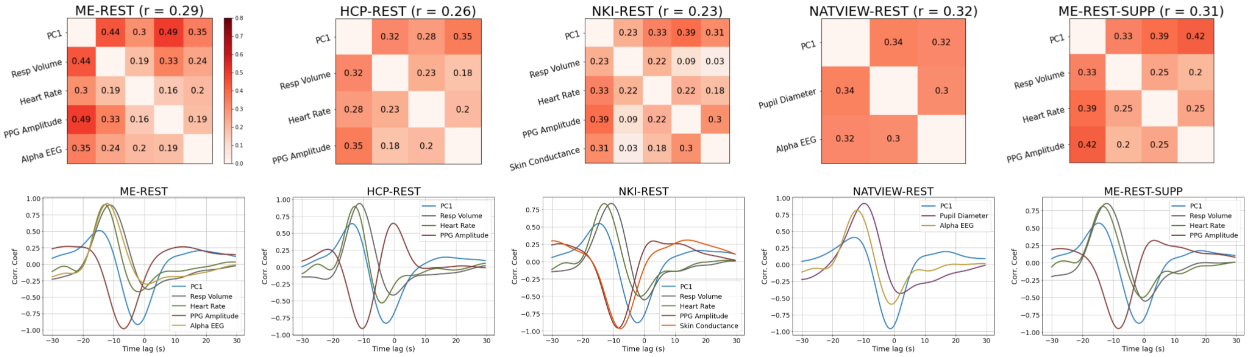

Supplementary Figure 10. **MCCA of Resting-State Datasets and Cross-Correlations. Top panel:** The pairwise correlations (top) between all physiological signals (including the global fMRI signal; PC1) in the first canonical component of the MCCA analysis and their time lags (bottom) for five resting-state datasets, as displayed in **Figure 1**. The average pairwise correlation is displayed beside the title of each correlation matrix. **Bottom panel:** To extract timing information between the signals in this low-dimensional space, we cross-correlated each physiological signal with its projection onto the first canonical component**.** The cross-correlations between each physiological signal and its projection onto the first canonical component are displayed in the bottom panel, where each signal is displayed in a different color. Comparison of the relative timing between peaks of the cross-correlation curves across

bioRxiv preprint doi: https://doi.org/10.1101/2023.01.19.524818; this version posted July 29, 2024. The copyright holder for this preprint (which was not certified by peer review) is the author/funder, who has granted bioRxiv a license to display the preprint in perpetuity. It is made available under aCC-BY 4.0 International license.

physiological signals provides the lead-lag relationships between signals within the first canonical component of MCCA.

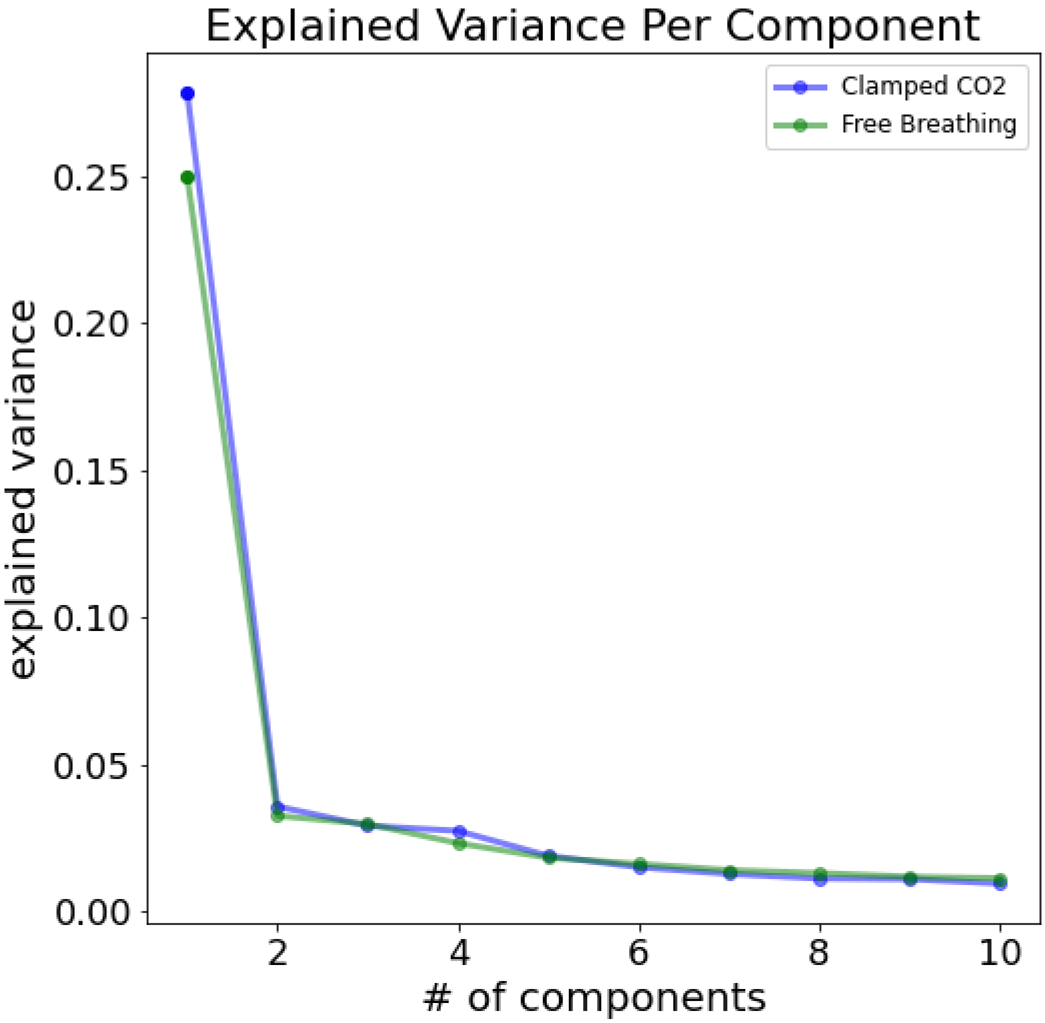

Supplementary Figure 11. **Scree Plot of Eigenvalues for Clamped CO2 and Free Breathing Conditions.** A scree plot of eigenvalues of the first ten components from PCA estimated from the free breathing (green) and 'clamped' CO2 (blue) condition. As can be observed, the spatial distribution of the global fMRI signal is maintained when variations of CO2 are experimentally suppressed. More generally, the low-dimensional spatial structure of fMRI time courses between the two conditions, as reflected in the scree plot, is similar between the two conditions.

bioRxiv preprint doi: https://doi.org/10.1101/2023.01.19.524818; this version posted July 29, 2024. The copyright holder for this preprint (which was not certified by peer review) is the author/funder, who has granted bioRxiv a license to display the preprint in perpetuity. It is made available under aCC-BY 4.0 International license.

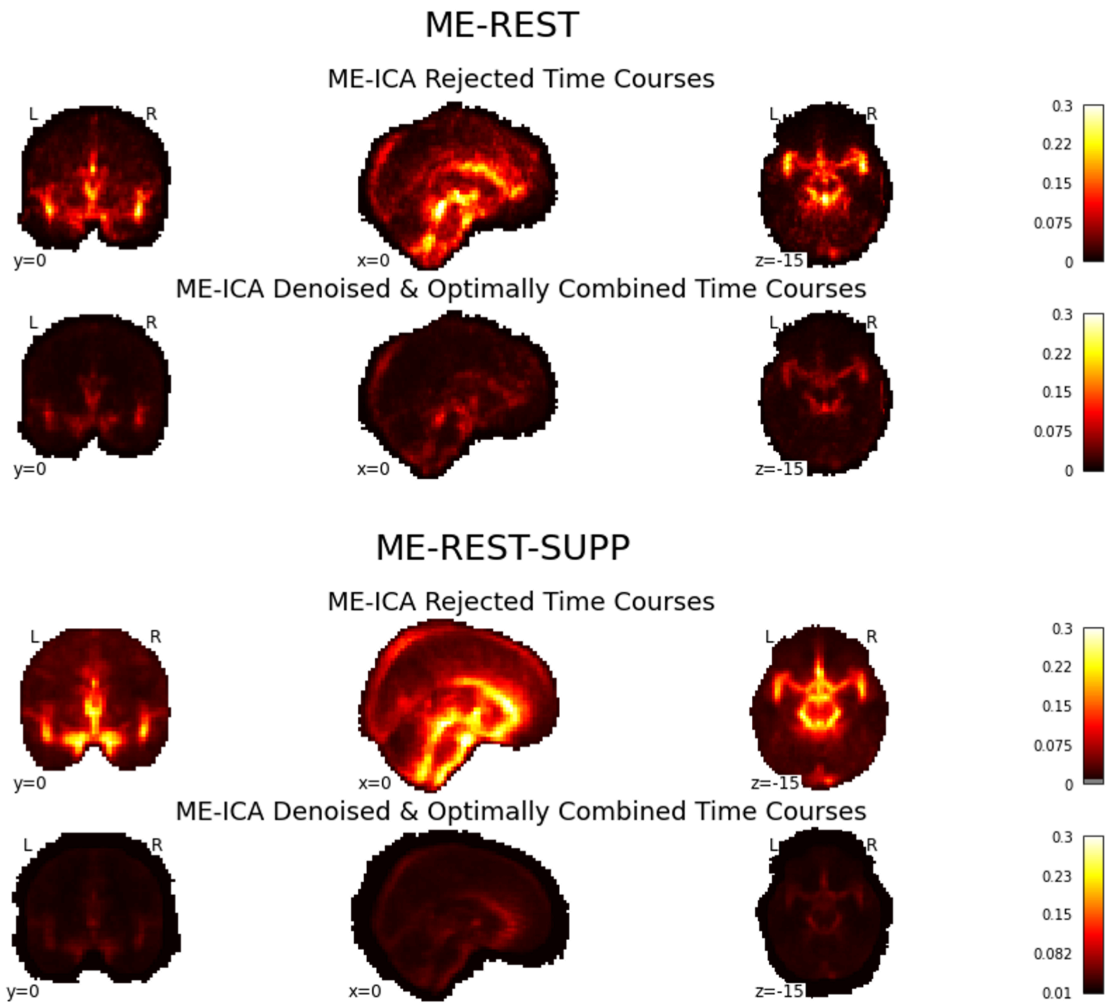

Supplementary Figure 12. **Explained Variance of High-Frequency Cardiac Regressors In Multi-Echo ICA Denoised Time Courses.** Brain maps of the group-averaged explained variance (R^2) estimates at each voxel by high-frequency cardiac (RETROICOR) regressors. To verify that the spatiotemporal pattern of the global fMRI signal (**Figure 3**) does not arise primarily from aliased cardiac pulsations, we performed regression modeling of (aliased) high-frequency cardiac RETROICOR regressors in Multi-Echo (ME) fMRI data. Specifically, we performed regression modeling on both the ME-ICA denoised time courses and on the time courses that were rejected as echo-time independent 'noise' by ME-ICA (i.e., time courses constructed from the rejected ME-ICA components). As can observed in the group-averaged R^2 maps, high-frequency cardiac pulsation effects are primarily observed in the ME-ICA rejected time courses, and are largely attenuated in the denoised time courses.
